# Effects of Ageing in Disinfectant Solution on the Corrosion Resistance and Antimicrobial Behavior of Copper Alloys

**DOI:** 10.3390/molecules28030981

**Published:** 2023-01-18

**Authors:** Florica Simescu Lazar, Marius Colin, Gaëlle Carré, Nicolas Bachelard, Jean-Paul Chopart, Sophie C. Gangloff

**Affiliations:** 1 Laboratoire Matériaux et Ingénierie Mécanique (MATIM), UFR de Sciences Exactes et Naturelles, Université de Reims Champagne-Ardenne, 51687 Reims, France; 2 Biomatériaux et Inflammation en site Osseux (EA 4691 BIOS), SFR CAP-Santé, UFR de Pharmacie, Université de Reims Champagne-Ardenne, 51095 Reims, France

**Keywords:** corrosion, copper alloys, antimicrobial activity, *Staphylococcus aureus*, XPS analysis, TOF-SIMS analysis

## Abstract

This work studies two copper-based alloys as potential antimicrobial weapons for sectors where surface hygiene is essential. Effects of different alloying elements addition at the same Cu content (92.5% by weight) on the corrosion resistance and the antibacterial performance of two copper alloys were studied in an aerated disinfectant solution (0.25% *v/v* Aniosurf Premium (D)) by electrochemical corrosion, X-ray photoelectron spectroscopy (XPS), time-of-flight secondary ion mass spectroscopy (ToF-SIMS) and antibacterial tests. Results showed that the nature of the alloying elements had a clear influence on the corrosion resistance and antibacterial performance. Electrochemical impedance results and surface analyses demonstrate the presence of organic compounds bound on the substrate and that a film covers part of the total active surface and may act as a protective barrier by preventing the interaction between metal and solution, decreasing the antimicrobial performance of copper-based materials. Low zinc and silicon contents in copper alloys allows for better aging behavior in D solution while maintaining good antibacterial performance. The XPS and ToF-SIMS results indicated that artificial aging in disinfectant enhanced Cu enrichment in the organic film formed, which could effectively stimulate the release of Cu ions from the surface.

## 1. Introduction

Healthcare associated infections (HAIs) are caused by a wide range of bacteria, fungi and viruses. HAIs frequently occur during medical treatment, especially in healthcare facilities, where they represent a concerning health issue [1,2]. They lead to longer hospital stays, higher readmission rates, higher care costs for patient with HAIs and higher mortality. Treating infections is becoming increasingly difficult given the increase in antimicrobial resistance and the decreasing number of effective antibiotics. A growing part of HAIs is caused by bacteria resistant to at least one type of antibiotic [2] and bacteria continue to accumulate resistance mechanisms. 

Bacterial contamination is extremely frequent in the patient’s environment. Medical devices, sinks or touch surfaces, such as doorknobs, tables, push plates, bed rails, door handles or handrails, can represent environmental reservoirs of microorganisms [3,4,5,6,7]. Worse still, pathogens responsible for HAIs, including methicillin-resistant *Staphylococcus aureus* (MRSA), *Acinetobacter* spp., vancomycin resistant Enterococcus (VRE), Clostridium difficile or noroviruses, can survive on these abiotic surfaces from few hours to several months if no disinfection process is done [3,8,9,10,11]. Several observations and experimentations suggest the important role of the environment in the dissemination and transmission of these pathogens and their indirect implication in HAIs [12,13,14]. 

Thus, touch surfaces represent a critical cross point to focus on. Commonly used surfaces (mostly made of plastic, stainless steel, coated metal or wood) are quickly recontaminated after being cleaned with common chemical disinfectants. For this reason, hospitals and establishments of accommodation for dependent older persons (EADOP) seek for surfaces which offer inherent antimicrobial properties and long-term protection against pathogens contaminating environment and institutions. The use of antimicrobial surfaces to lower microbial contaminations, added to common infection prevention practices, might reduce patient’s risk of exposure to pathogens [13,15]. Such strategies could be particularly beneficial for the most vulnerable patients, such as those in the intensive care units (ICU) and in EADOP.

Copper alloys have recently attracted attention as a very promising antimicrobial weapon for areas where surface hygiene is paramount [16,17,18,19,20,21]. Currently, antimicrobial mechanisms of metallic copper remain incompletely understood; however, numerous studies have shown that a wide range of viruses, fungi and bacteria, including *Staphylococcus aureus*, are inactivated within a few hours of exposure to copper containing surfaces [3,22,23,24,25,26]. Following these observations, several studies were conducted in healthcare facilities to validate the concrete efficiency of copper alloys as antimicrobial touch surfaces [5,7,26,27,28]. In these investigations, copper equipped areas were compared to non-copper areas, both situated in the same facilities and numerous bacterial samplings of surfaces were conducted. Results from the majority of these studies demonstrated significantly lower bacterial contaminations on copper surfaces, revealing the impact of copper on the microbial risk in healthcare facilities. However, Colin et al. [3] revealed a potential progressive loss in the antibacterial efficiency of copper door handles through years of use and disinfection. 

The strong antimicrobial activities of copper alloys containing more than 90% of copper are due to copper ion releasing from the surface through oxidation [29,30] and, therefore, this oxidation is essential. However, daily use and disinfection of the touch surface may lead to important modification of the alloy surface. Indeed, a certain disadvantage in contact surfaces composed of materials based on copper is their susceptibility to corrosion during cleaning and environmental disinfection, which can ultimately result in an alteration of antimicrobial properties [3,31]. As a result, producing copper alloys with optimal and time-stabilized antimicrobial activities is a prerequisite and represents an indispensable step in the development of these device surfaces.

The aim of the present study was to investigate the effect of aging in aerated 0.25% *v/v* Aniosurf Premium solution (D) on different copper alloys structures and antibacterial function. More precisely, two alloys namely, Cu_92.5_X where X is Al_c_Ag_d_ or Zn_a_Si_b_ for “Former alloy” and “New alloy”, respectively, and commercially pure copper (Cu), have been investigated to assess the dynamics of the antimicrobial properties of these two copper alloys on *Staphylococcus aureus*, a pathogen which is frequently involved in HAIs. Meanwhile, the electrochemical experiments were used to characterize the corrosion properties. The adsorption film structure on the surface of the different copper-based samples were also analyzed by XPS and ToF-SIMS techniques [32]. Hence, the effects of adding different alloying elements at the same copper content on the mechanism of corrosion processes taking place at the electrode/solution interface were discussed. Several copper-based materials were studied according to their corrosion and antibacterial performances following contact with an aggressive disinfection medium. It is believed that this work is helpful to provide a profound research base for further development of this novel class of antibacterial copper alloys.

## 2. Results and Discussions

The spread of bacterial agents is an accurate problem, notably through inanimate surfaces contamination [8,9,12]. Indeed, these surfaces can play the role of microbial reservoir, negating the effect of hand washing, which currently remains the essential step against the dissemination of pathogens. Given this context, the use of surfaces that have residual antimicrobial activities brings a new perspective for constant and inherent disinfection. Nevertheless, in medical environment, the surfaces are regularly disinfected; therefore, it is necessary to appreciate the potential adverse effects of disinfectant solution on surfaces, such as copper and copper alloys.

### 2.1. Corrosion Tests

#### 2.1.1. Open Circuit Potential Measurements 

**Figure 1:**
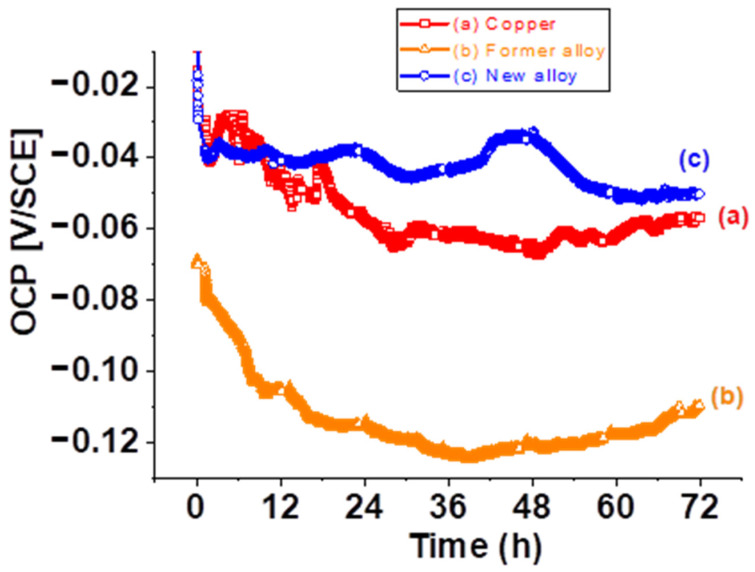
OCP profiles of selected Cu based samples aged in D solution: (**a**) Copper, (**b**) Former alloy and (**c**) New alloy.

The open circuit potential (OCP) profiles of copper-based samples aged in Disinfectant solution during 72 h are shown in Figure 1. For the pure copper sample, the OCP started at approximately −0.006 V/SCE, then rapidly decreased and stabilized at around −0.060 V/SCE after ∼24 h. This curve is similar with OCP profile obtained by Li et al. in concentrated chloride solution [33]. OCP of the Former alloy (Figure 1b) start of ~−0.07 V/ECS and stabilize after 24 h at −0.110 V/ECS. The New alloy shows a similar behavior to that of pure copper. Thus, for this sample, the OCP decreases rapidly from 0 V/ECS and stabilizes after 12 h of immersion at a potential greater than that of pure copper. This curve is smoother than that of pure copper. Its evolution corresponds to an active state of the samples. For the New alloy, the decrease in OCV values is slightly higher than those of pure copper. After 72 h of immersion in D solution, the New alloy exhibited more noble open circuit potential (~+60 mV) than the Former alloy as presented in Figure 1. The comparison of these results with OCV evolution measured for Former alloy shows that the copper-based samples aged in D solution are subject to corrosion following theirs composition [34]. It seems that adding Zn/Si elements to pure copper allows a better electrochemical behavior during aging.

#### 2.1.2. Electrochemical Impedance 

The electrochemical impedance diagrams (EIS) at the OCP are obtained by EIS measurements at 12 h, 24 h and 72 h, respectively, because after 12 h immersion in disinfectant all the OCP profiles show a constant potential during 30 min (time of impedance measurement). 

**Figure 2:**
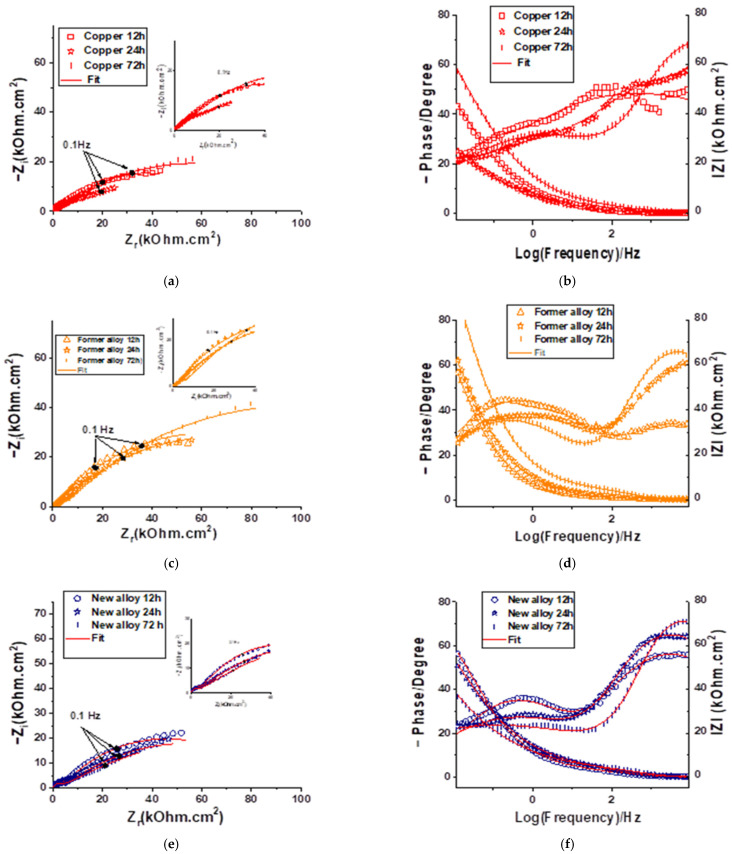
EIS measurements corrected by resistance of electrolyte (R_e_) of copper-based samples at OCP during immersion in D solution. Nyquist plots for: Copper (**a**), Former alloy (**c**) and New alloy (**e**). Bode plots for: Copper (**b**), Former alloy (**d**) and New alloy (**f**).

A comparison between the impedance spectra Nyquist plots and Bode diagrams for the Copper, Former and New alloys are presented in Figure 2a,c,e and Figure 2b,d,f, respectively. All these EIS diagrams were corrected by electrochemical resistance of electrolyte (EIS-R_e_). The shapes of the Nyquist impedance spectra show two relaxation time constants (two capacitive loops). This result is also indicated by the two peaks in the phase angle plot at the high (HF) and low frequencies (LF). 

For the Copper and Former alloy, the magnitude of the total impedance enhanced after 72 h immersion in D solution. This value is lower and does not change with the duration of immersion for the New alloy in the same conditions.

The phase angle diagram evolution, with immersion time shows that the presence of chemical compounds in the D solution simultaneously results in a competition between the formation and local destruction of the film formed on polished surface during the first hours of immersion. Inspection of the Bode plots (Figure 2b,d,f) shows that the two capacitive loops are similar at 12 h for the Cooper and New alloy samples, then the high frequencies loops increase simultaneously with the decrease in low frequencies loops. For the Former alloy, the evolution is the same, except that at 12 h of immersion the phase corresponding to the high frequency region is very weak. This result suggests that the adsorption film formed on the samples surface is different and depends on Cu based alloy composition.

**Figure 3:**
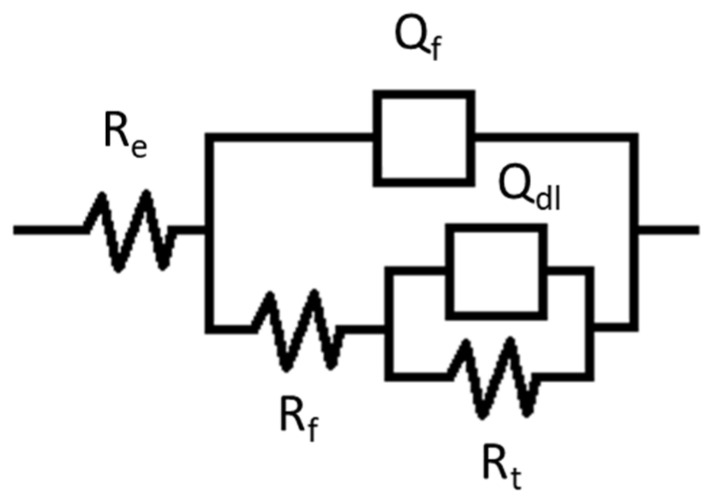
Electrical equivalent circuit used to fit the impedance data shown in Figure 2.

The electrical parameters obtained by fitting EIS data with two-time constants electrochemical equivalent circuit shown in Figure 3 are listed in Table 1. The electrochemical equivalent circuit (EEC) comprises two constant phase elements (CPE) connected in series with the uncompensated resistance (R_e_) which represents the electrolyte resistance and the resistance due to the electrical connections [35]. The R_ct_ represents the charge transfer resistance of copper bases samples dissolution, between the metal surface and the Helmoltz plane (OHP) to the metal—solution interface. The Nyquist plots show an apparent deviation from the ideal semicircle which is explained by the inhomogeneity of the surface due to the roughness, impurities and the adsorption of chemical compounds. In this situation, a constant phase element (CPE) and a proportionality factor (Q) are commonly used in place of an ideal capacitor to model the EIS diagrams. Q_dl_ is the constant phase element present at the metal–solution interface while the Q_f_ presents the CPE of the surface film and R_f_ the corresponding film resistance. The goodness of fit values (χ^2^) is of the order 10^−4^.

For the Copper and Former alloy 72 h immersion in Disinfectant solution, leads an increase in R_t_ up to 190 kΩcm^2^. This suggests that –NH_2_, –SH groups are adsorbed at the metal surface and protect it. Q_f_ parameter decreases for all the samples during 72 h of immersion in D solution, but it seems that the rate of reaction is different for each sample. Thus, we note a less important evolution of the film for the New alloy. The electrochemical behavior of the metal shows a constant evolution for this last sample. At 72 h, it seems that the Q_f_ values of organic film is similar for all the samples. This result shows that the thickness of the formed film is the same, but the active surface is different for the New alloy. It is noted that the Copper film resistance measured at 12 h of immersion is more efficient than that observed after 72 h of immersion. Therefore, the film resistance, R_f_ was 10.6 kΩcm^2^ and decreases to around 2.2 kΩcm^2^ after 72 h in the presence of D solution. This behavior can be attributed to the diffusion of different anions into the protective oxide layer reacting with ions to form a non-adherent copper corrosion product. This diffusion process which disturbs the double layer at low frequency [35] can be responsible for the low value (around 0.5) of the n_dl_ parameter. The resistance of barrier film formed on the Former alloy after 12 h in D solution was 1.5 kΩcm^2^ and increases slowly to 5 kΩcm^2^ at 72 h. This can be attributed to the presence of aluminum in this alloy which generates a more compact oxide film.

In contrast, for the New alloy, the magnitude variation of the total impedance and film resistance were weak. This behavior can be explained by the presence of zinc and silicon in this new material, which seems less protected by the oxides film generate after 72 h of immersion in D solution.

### 2.2. XPS Study

The X-ray Photoelectron Spectroscopy analyses (XPS) were performed on sample surfaces after 0 h, 2 h, 12 h, 24 h and 72 h in D solution prepared according to the Section 3.1. protocol. The XPS data were used to obtained a quantitative elementary analysis and a study of the chemical forms of the detected elements.

#### 2.2.1. Elementary and Quantitative Analysis

**Figure 4:**
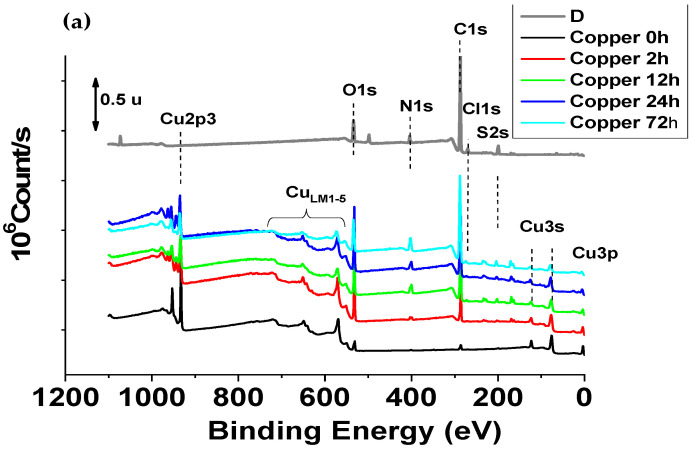

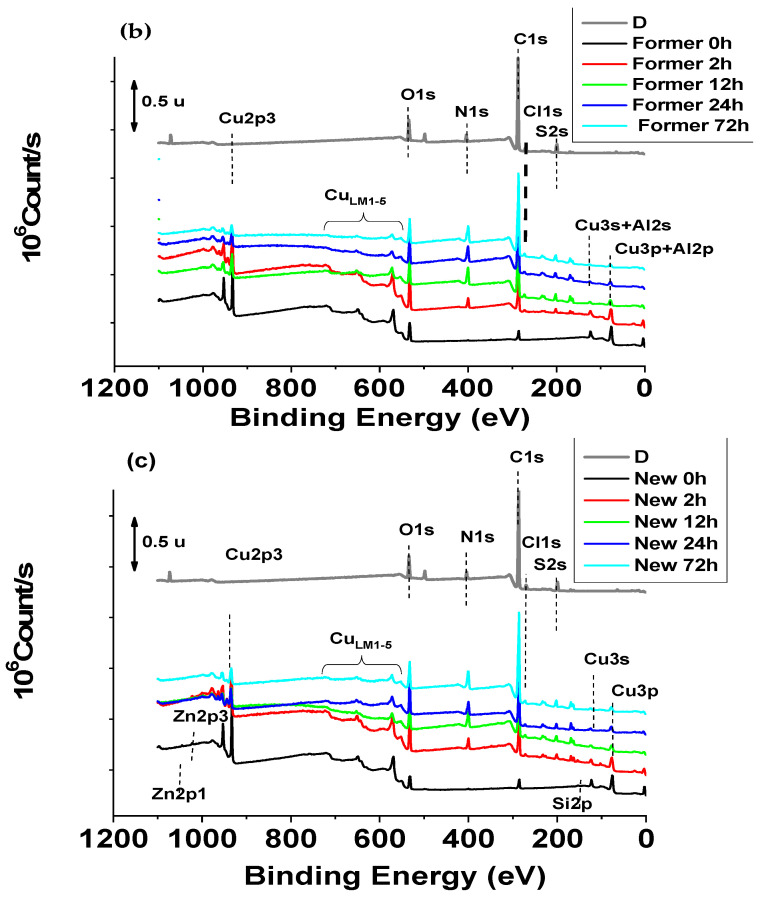
Survey XPS spectra for samples Disinfectant on a silicon substrate (D/Si substrate) and copper-based materials after 0 h, 2 h, 12 h, 24 h and 72 h immersion in D solution: (**a**) Copper, (**b**) Former alloy and (**c**) New alloy. Only major elements are reported on the spectra.

XPS analyses were performed on surface samples immersed for 0 h, 2 h, 12 h, 24 h and 72 h in D solution. The survey XPS spectra (Figure 4) are principally composed of C, O, Cu, N, Cl, S. Zn, Si and Al were observed at the corresponding samples surface exposed during 2 h in D solution. Additionally, for the New alloy 2 h sample small peaks of Zn2p1, Zn2p3 and Si2p were detected. The at% of Al at the surface of Former alloy 2 h was calculated from the Cu_3s_ peak by subtracting the contribution of the Cu_3s_ peak. This contribution was estimated by the ratio of the Cu_3s_/Cu_2p3/2_ area peaks with a pure copper. No other element was observed, within the limit of the technique sensitivity (0.1 to 0.5% at.). The elementary compositions (expressed in atomic% (at%) and obtained from high resolution spectra) are summarized in Table S1. An analysis of the disinfectant applied to a silicon substrate (noted D/Si substrate) was also conducted. 

#### 2.2.2. Chemical Forms of Detected Elements 

The attributions of the chemical forms are conducted according to internal references, the “Handbook of X-ray Photoelectron Spectroscopy” [36] and to an open XPS database [37]. All the detected peaks have been readjusted with respect to the theoretical position of the C_1s_ peak for the C-C and C-H groups in the aliphatic compounds at 285 eV. Differential charge effects have been noted for some samples, which makes difficult the chemical forms interpretation (doubling of the peaks). These samples were named Copper 12 h, Copper 72 h, Former alloy 72 h and New alloy 24 h and highlighted with “*” in the chemical form decomposition tables.

- Carbon: Peak C 1s

The carbon peak C_1s_ has four corresponding components: (1) C-C/C-H bonds (for aliphatic compounds at 285 eV), (2) C-O bonds at 286.4 eV, (3) O-C-O and/or C = O bonds at 287.8 eV and (4) O-C = O bonds around 289 eV.

The proportions of the different chemical forms of carbon, obtained by the decomposition of the C_1s_ peak (expressed as a percentage of form relative to the total area of the peak), are given in Table S2.

- Oxygen: Peak O_1s_

The oxygen O_1s_ peak is large and has several elements (binding energy) corresponding to metallic oxides, minerals and O/C bonds.

- Copper: Cu _2p3/2_ and Cu _LMM_ peaks

The Cu _2p3/2_ peak of copper has several components. One centered at 932.7 eV, corresponding to the metal form (copper with an oxidation degree of 0) and/or Cu_2_O and/or Cu_2_S (oxidation degree of +1). Table S3 shows the proportion of chemical forms of copper in the samples. The study of the Auger peak makes it possible to distinguish these two forms. For samples polished (noted 0 h), whatever the used metallic material, the metal form is mostly detected. For the other samples, the Auger peak is located for a kinetic energy between 915.7 eV and 916.7 eV, which corresponds to the form Cu_2_O and/or Cu_2_S (oxidation degree +1). Another component, centered around 934–935 eV with a satellite peak at 943.7 eV, corresponding to forms with oxidation degrees +2 (CuSO_4_, CuO, CuCl_2_, …) has been also detected.

- Nitrogen: Peak N_1s_

The N_1s_ peak of nitrogen has three components (Table S4). The first peak is detected around 399–400 eV and was attributed to organic forms. The second, measured around 398 eV, corresponding to other organic forms, even to organometallic forms (no references for this type of compound in the tables) or cyanide. The last component, corresponding to an ammonium and/or nitroso form (-NH3, N=O) was unregistered at ~402 eV.

The proportions of the different nitrogen chemical forms, obtained by the decomposition of the peak N_1s_ (expressed as a percentage of form relative to the total area of the peak), are given in Table S4.

- Chlorine: Peak Cl_2p_

The chlorine peak Cl_2p_ shows least two components: (a) one at 198 eV, corresponding to a chloride form and (b) another at 200.5 eV, corresponding to an organic form (Cl-C bond).

The proportions of the different chemical forms of chlorine, obtained by the decomposition of the Cl_2p_ peak (expressed as a percentage of form relative to the total area of the peak), are given in Table S5.

- Sulfur: Peak S_2p_

The sulfur peak S_2p_ shows two components: (a) one (around 162 eV), corresponding to a sulfide form and another (at ~168–169 eV), corresponding to sulphate and/or sulphonate forms. The proportions of the different chemical forms of sulfur, obtained by the decomposition of the peak S_2p_ (expressed as a percentage of form relative to the total area of the peak), are given in Table S6.

We note that the aluminum, zinc and silicon elements are present and have content that is too low or have a chemical shift that is too low to determine their chemical environment.

These XPS results make it possible to highlight a systematic increase in the carbon content from two hours of immersion in the disinfectant for all samples. This increase is probably linked to the formation of an organic layer on the surface.

Moreover, an evolution of the chemical forms of copper with the formation of species at oxidation degree of +1 (Cu_2_O and/or Cu_2_S) and of +2 (CuSO_4_, CuO, CuCl_2_ …) which is difficult to distinguish or to assign between samples. The metal form of copper is no longer detected after 12 h of immersion but given the “covering” and the weakest detection of it, it is difficult to say whether it is an oxidation or the formation of copper complex or copper organometallic compounds. Nitrogen has been identified on all samples, in several forms (ammonium, organic and/or organometallic). The nitrogen content increases sharply with the exposure time in the disinfectant solution. The proportion of ammonium form increases from “2 h” to “72 h”.

Chlorine is also detected on all samples and is in chloride form for the “0 h” samples. After 2 h of immersion in D solution, the chlorine content decreases and an organic form (C-Cl bonds) is observed. The proportion of organic form tends to increase with the time of “soaking” in the D solution. Sulfur is detected as soon as the alloy has been brought into contact with the disinfectant. Sulfur was not expected. An analysis of the disinfectant solution deposited in a thin layer on a neutral substrate (silicon wafer) showed the presence of sulfur traces in the sulfide and sulphate form in the solution. Sulfur is also detected in the sulfide and sulphate form on the copper-based samples.

The results obtained by XPS analysis made it possible to build an affinity between the D solution and the samples based on copper, which results in a “covering” of the surface (or the formation of a film on the surface). The film formation generates: (a) an increase in the carbon content, (b) an increase in the nitrogen content (presence of a larger ammonium form with the exposure time in the solution), (c) the presence of chlorine (more of organic and/or organometallic forms) and the presence of sulfur (in sulfate and sulfide form). The sulfur being present in trace amounts in the D solution.

The differences between the composition and/or the chemical forms revealed by the XPS analyses do not allow to clearly explain the differences in electrochemical behavior measured by EIS for copper-based alloys. To provide more information on the species formed at the samples extreme surface and, in particular, the species possibly formed with copper, zinc, silicon and aluminum, the XPS results were completed by a ToF-SIMS analysis of the polished samples (noted 0 h) and immersed for 72 h in D solution.

### 2.3. ToF-SIMS 

#### 2.3.1. ToF-SIMS Analysis of D Solution Deposited on an Aluminum Substrate

According to the product notice [38], the disinfectant solution consists of: (1) didecyldimethylammonium chloride (82 mg/g), (2) chlorexidrine digluconate (5 mg/g), (3) polyhexamethylene biguanide hydrochloride (0.24 mg/g) and (4) excipients.

The ToF-SIMS analyses performed on the disinfectant deposited on aluminum substrate showed the presence, in this disinfectant, of inorganic and organic compounds.

The inorganic compounds identified are: Na (intense), Al and Al_w_H_y_O_z_ (for the metallic elements coming from the substrate, sulfur species S, SH, S_2_, CSN and SO_x_ (intense SO_3_ and HSO_4_), PO_x_ phosphorus species, CN and CNO nitrogen species (in negative ions), NO_x_ nitrogen species (mainly NO_2_ and NO_3_), Cl (intense).

The organic compounds detected are: C_x_H_y_^+^ hydrocarbon species (in positive ions) and C_x_/C_x_H^−^ carbon clusters (in negative ions), the oxygenated organic species of general formula C_x_H_y_O_z_ with in particular the molecular ions of linear fatty acids in negative polarity and ions compatible with the fragmentation of Digluconate (C_2_H_3_O_2_^−^, C_3_H_5_O_3_^−^, C_3_H_5_O_4_^−^, C_6_H_11_O_7_^−^ (M-H)^−^) [38] the nitrogenous organic species with general formula C_x_H_y_N and/or C_x_H_y_N_z_ (C_3_H_8_N^+^, C_12_H_26_N^+^ intense) particularly with the presence of Didecyldimethylammonium (C_22_H_48_N^+^), other positive ions at 452 uma, 464 uma; C_6_H_4_N^−^, C_7_H_5_N_2_^−^ ions, which can be derived from the fragmentation of chlorhexidine; the C_2_N_3_^−^ ion which can come from the fragmentation of chlorhexidrine and/or polyhexamethylene biguanide; and five unidentified positive ions at 635 uma, 687 uma, 1139 uma, 1170 uma and 1202 uma (Table S7).

#### 2.3.2. ToF-SIMS Analysis of Copper Based Materials before and after Immersion in D Solution 

Tables S8–S10 show the relative intensities of a selection of detected compounds on the samples surface. The relative intensities are obtained by normalizing the species peak area with copper ion peak area of the sample matrix brought to 100% and considered as characteristic of the substrate. In these tables, all the values are therefore percentages with respect to the area of the characteristic ion of the matrix with the exception of the value indicated in the matrix column which corresponds to the area of the characteristic ion of the matrix. Note that for each spectrum produced (three per polarity and per sample), the area of the characteristic ion of the matrix is different because the surface contaminations more or less, mask the substrate.

##### ToF-SIMS Analysis of Polished Copper (Copper 0 h) Sample

The inorganic compounds identified on the surface of the polished copper (Copper 0 h) during ToF-SIMS analysis are: intense copper in oxide form (Table S8) and the presence of copper chloride ions (Table S10).

The organic compounds found on this same sample are: C_x_H_y_^+^ hydrocarbon species (in positive ions) and C_x_/C_x_H carbon clusters (in negative ions). These species (Table S9) are formed from the fragmentation process of any type of organic molecule containing a hydrocarbon structure. Their presence is probably due mainly to sample surface contamination during sample preparation.

##### ToF-SIMS Analysis of Polished Former Alloy 0 h

The following inorganic compounds has been detected on the Former alloy extreme surface: intense copper peak in oxide form Al and Al_w_H_y_O_z_ for the metallic elements (Table S8), and the presence of copper chloride ions (Table S10).

The organic compounds identified in same time are: C_x_H_y_^+^ hydrocarbon species (in positive ions) and C_x_/C_x_H^−^ carbon clusters (in negative ions). These species are formed from the fragmentation processes of any type of organic molecule containing a hydrocarbon structure and their presence is probably due to the sample contamination (Table S7).

##### ToF-SIMS Analysis of Polished New Alloy 0 h

The inorganic compounds identified on the New alloy 0 h surface are: intense copper in oxide form, presence of copper chloride ions and Si in oxide form (Si_w_H_y_O_z_).

The following organic compounds has been identified: C_x_H_y_^+^ hydrocarbon species (in positive ions) and C_x_/C_x_H^−^ carbon clusters (in negative ions). These species are formed from the fragmentation processes of any type of organic molecule containing a hydrocarbon structure (Tables S7–S9). The oxygenated organic species of general formula C_x_H_y_O_z_ with in particular the molecular ions of linear fatty acids in negative polarity. 

#### 2.3.3. Surface Composition of Copper, Former Alloy and New Alloy after 72 h Immersion in D Solution

The ion mass spectra of pure copper, the Former alloy and the New alloy after 72 h immersion in D solution are similar. For inorganic compounds we note the presence of intense copper peak in oxide form, presence of copper chloride and copper sulfate ions.

**Figure 5:**
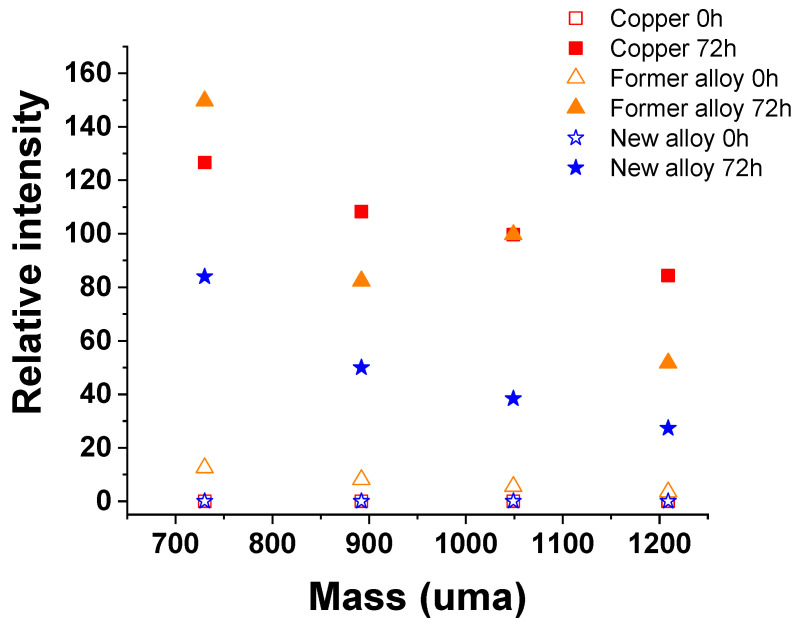
Comparison of Cu_x_Cl_y_S_w_O_z_(CN)_r_ type ions mass spectra obtained on copper-based materials before and after 72 h immersion in D solution.

In the high molecular weights, we observe massive peaks that can correspond to Cu_x_Cl_y_S_w_O_z_ (CN)_r_ type ions, Si and Al, for metallic elements (Table S8), Sulfur species S, SH, CSN and intensely SO_x_, peak CN and CNO nitrogen species (in negative ions), NOx nitrogen species (mainly NO_2_ and NO_3_) and Cl (intense) (Table S10). Figure 5 shows a comparison of Cu_x_Cl_y_S_w_O_z_ (CN)_r_ type ions mass spectra obtained on copper-based materials before and after 72 h immersion in D solution. 

After 72 h immersion in D solution all the samples present these compounds. The new alloy appears to form these compounds in smaller quantities or the formation rate of these products is slower.

The organic compounds detected are: C_x_H_y_^+^ hydrocarbon species (in positive ions) and C_x_/C_x_H carbon clusters (in ions negative), the oxygenated organic species of general formula C_x_H_y_O_z_ with in particularly the properties of linear fatty acids ions with negative polarity and compatible ions with the fragmentation of Digluconate (C_2_H_3_O_2_^−^, C_3_H_5_O_3_^−^). We note, the absence of the digluconate molecular peak detection and in addition, the presence of positive ions not identified at 635 uma, 1170 uma and 1202 uma (Table S7).

This semi-quantitative approach (Tables S8–S10) mainly highlights that the samples can be classified as follows: from the one with the most intense substrate to the one with the least intense for Copper 0 h that Copper 72 h, Former alloy 0 h that Former alloy 72 h and for New alloy 0 h that New alloy 72 h. 

The samples immersed during 72 h in D solution clearly show the ions from the solution D: sulfur ions including ions linked to copper (Cu_x_Cl_y_S_w_O_z_(CN)_r_), chlorine, hydrocarbon ions and nitrogenous hydrocarbons bound to chlorhexidine and/or to polyhexamethylene biguanide. 

The ToF-SIMS analyses of the copper alloys (Former alloy and New alloy) at 0 h and 72 h lead mainly to the same observations as those obtained on the pure Copper.

The polished samples present intensely the substrate peak (copper ions and copper oxide ions) and include some organic contamination by linear fatty acids. 

The Copper 72 h sample are characterized by the presence of species from solution D and the interaction of sulfate ions, chlorine, nitrogen, solution D with copper (appearance of peaks, such as Cu_x_Cl_y_S_w_O_z_ (CN)_r_).

We note in particular, for all the samples immersed in D during 72 h, very similar spectra for the same contact time. In addition, ToF-SIMS now allows clear identification of different species on the surface and provides conclusive evidence of whether certain species are present or not [39,40]. Indeed, extreme surface chemistry is very comparable and is mainly linked to the deposition and/or interactions between elements of D solution with copper (predominant element in the three samples). Compounds, such as those based on Zn …, Si … and Al …, it is the only differences between studied samples.

Regarding these results and EIS measurements it can be noted that the Zn and Si based copper compounds formed a less protects film and leaves more active surface for the New alloy. 

### 2.4. Antimicrobial Performance of the Copper Surfaces

As seen previously, copper alloy handles in EADOP preserved their antibacterial activity over more than three years of use. In the in vitro assays against MRSA, the efficiency of these handle copper surfaces seems to slowly decrease with time. While unused copper door handles allowed an average 3.2 logs reduction in number of MRSA within two hours, the reduction dropped to 2.7 logs after one year and 1.7 logs after three years of use [3]. In the present study, the effect of accelerated aging with disinfectant was evaluated on two different alloys.

**Figure 6:**
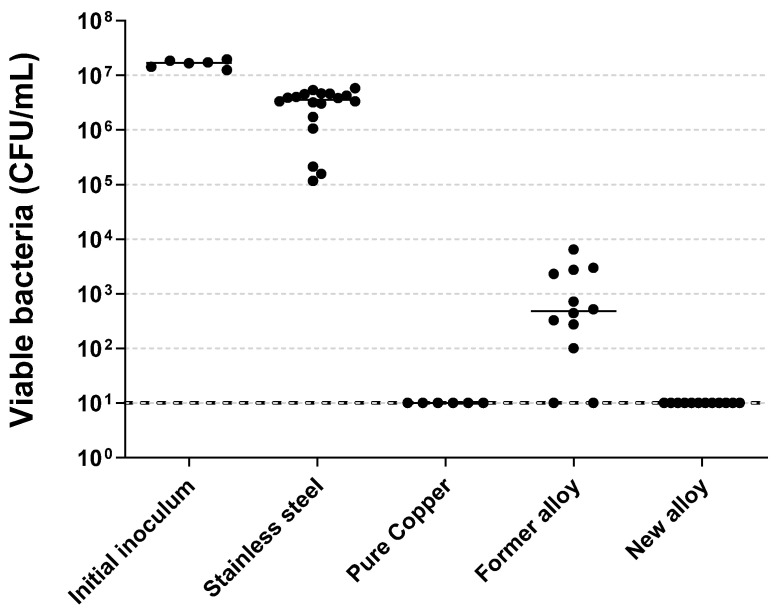
Quantity of viable MRSA recovered after a contact of two hours with metallic samples. Black lines represent medians. Every condition demonstrated significant differences to each other, except “Pure Copper” vs. “New alloy”.

First, the antibacterial activities of the two copper alloys were evaluated and compared to SS and pure copper (Figure 6).

SS coupons demonstrated slight punctual variations in the number of the remaining viable bacteria. Compared to SS, the Former alloy demonstrated a bacterial burden reduction ranging from 2.8 to 5.6 log. The New alloy and pure copper showed a higher and optimal bactericidal activity, with bacterial burdens reduced below the threshold of 10 CFU/mL, which corresponds to reductions of at least 4 log in the number of bacteria recovered as compared to SS. The not aged New alloy was as efficient as pure copper against MRSA.

As before with corrosion, to apprehend the impact of time in use on the antibacterial activity, the copper and copper alloys were artificially aged in D solution.

**Figure 7:**
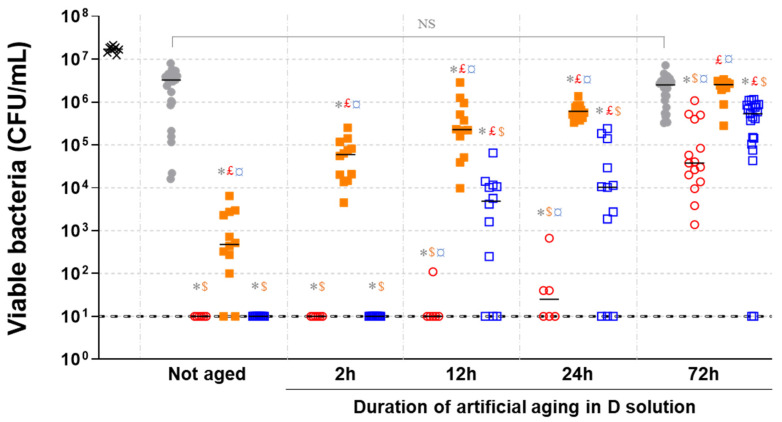
Quantity of viable MRSA recovered after a contact of two hours with artificially aged metallic samples. × Initial inoculum. 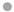 Stainless steel. 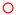 Pure copper. 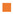 Former alloy. 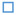 New alloy. Black lines represent medians. Symbols indicate significant differences compared to: * Not aged stainless steel; £ Pure copper for same aging; $ Former alloy for same aging; ¤ New alloy for same aging. NS: Not significant.

Figure 7 shows quantity of viable MRSA recovered after two hours contact with artificially aged metallic samples.

No significant difference appeared when comparing the aged and not aged SS coupons. Conversely, for the three copper-based samples, the remaining bacterial burdens were higher on artificially aged samples mostly after 12 h. On the not aged Former alloy, as on previous assay (Figure 6), some bacteria were persistent (between 10^2^ to 10^4^ CFU/mL), while no viable bacteria had been detected on the New alloy and on pure copper surfaces. After 2 h aging, the quantity of surviving bacteria increased on the Former alloy, with up to more than 10^5^ CFU/mL (equivalent to the reduction of 1.5 log of the initial bacterial burden). For the New alloy and pure copper, the antibacterial activity was as strong as not-aged samples without any viable bacteria detected. After 12 h of aging, the number of viable bacteria was even higher on the Former alloy (exceeding 10^6^ CFU/mL for some samples) and some viable bacteria were detected on the other samples. The presence of bacteria was observed in the majority of the deposit spots on the New alloy and only on one spot on pure copper. After 24 h, for each condition, the quantity of viable bacteria recovered was higher than at 12 h. Results on the Former alloy were particularly reproducible with a mean at 2 × 10^5^ CFU/mL. The number of bacteria recovered on the New alloy were more variable with some values exceeding 10^5^ CFU/mL, while some others were still below the threshold (10^1^ CFU/mL). Concerning pure copper, the number of viable bacteria remained low with a mean around 10^2^ CFU/mL. After 72 h of immersion in D solution, all copper alloys showed reduced antibacterial activities. For SS, pure copper, Former alloy and New alloy median values of viable bacteria were 3 × 10^6^ CFU/mL, 4 × 10^4^ CFU/mL, 3 × 10^6^ CFU/mL, and 5 × 10^5^ CFU/mL, respectively. For an aging of 72 h, no significant differences in the number of viable bacteria were observed between SS and Former alloy. Conversely, the New alloy and pure copper have almost the same residual antibacterial activity. 

The results obtained here clearly demonstrate the progressive decrease in antibacterial efficiency of every copper-based material when in contact with the D solution. Former and New alloy displayed very different responses, the first quickly losing the antibacterial activity, while the second conserved an optimal activity after a 2 h treatments. For each immersion time evaluated, the antibacterial activity of the New alloy remained better than the Former alloy. Thus, the sensitivity of an alloy toward disinfectant treatments is highly impacted by the material composition.

### 2.5. Correlation between EIS, XPS, ToF-SIMS Results and Antibacterial Activity

Both surface analyses methods (XPS and ToF-SIMS) revealed the presence of sulfur in the D solution and, consequently, the organic sulfur products formation on the copper-based alloys surface. Barbouth et al. [41] showed that chemisorbed sulfur can modify the electrochemical behavior of copper in an acid medium. Its presence results in a good slowdown in metal attack speed. These authors attributed the cathodic protection of copper by sulfur to the formation of a copper-sulfur compound characterized by a potential less noble than that of copper. Moreover, Dilimon et al. [42] found that the self-assembly of normal and chelating alkanethiols (Sulfur based organic compounds) on copper surface is a fast adsorption step followed by the long-term additional adsorption and consolidation of formed sulfur based organic films. These previous studies prove that copper is a sensitive material to the sulfur presence. Organic molecules containing sulfur can easily link to its surface and form a film that protects it against corrosion but prevents direct contact between the metallic surface and bacteria, thus avoiding an antibacterial action of copper ions [29]. The results obtained by EIS, XPS and ToF-SIMS show that the New alloy composition is less favorable to the formation of this film. This phenomenon gives to this new material a better ability to remain efficient against bacterial contamination, even under repeated contact with not adapted disinfectant solution. 

## 3. Materials and Methods

### 3.1. Chosen Copper Alloys and Material Preparation for Corrosion Tests

Copper alloys samples measuring 4 cm × 0.8 cm × 0.8 cm were provided by Lebronze alloys Group, Suippes, France. During material elaboration, aluminum (Al) and silver (Ag) has been added at 92.5 wt% pure copper (Cu) for alloy noted “Former alloy”. The second alloy, noted “New alloy”, has been obtained by addition of 7.5 wt% zinc (Zn) and silicon (Si) for the same Cu content. Their exact chemical compositions remain confidential. As a result of the extensive experience acquired over more than 80 years, the composition of the copper alloys that were produced by LBA makes it possible to obtain materials with good metallurgical properties with exceptional ductility and elongation rate together with good mechanical properties. The compositions of these alloys enable good cold formability and complex shapes, they also allow the design of components that would not hurt their environment if they were to fail. Moreover, they are non-magnetic and resist mechanical wear and galling. Commercial grade copper (99.9% Cu) was obtained from GoodFellow. All corrosion tests were conducted on the commercial grade copper, which were Former and New copper alloys. Before each test, all specimens were mechanically polished using successive grades SiC papers down to 1200 grit, then cleaned and rinsed with deionized water and finally dried for one hour. Immediately after this preparation, the electrochemical measurements were done in naturally aerated 0.25% *v/v* ANIOSURF Premium Disinfectant solution (D) at ambient temperature. Aniosurf Premium (Laboratoire Anios, Lezennes, France) was selected as it is widely used as disinfectant for touch surfaces in French healthcare facilities and at a percentage 0.25% *v/v* Aniosurf in the cleaning procedures [3]. 

### 3.2. Electrochemical System/Corrosion Tests

Electrochemical experiments including impedance spectroscopy were performed in a three-electrode electrochemical cell of 250 mL, using a PGZ 100 RADIOMETER Potentiostat/Galvanostat and VoltaMaster software 4. Copper based materials embedded in epoxy resin with an exposure area of 1 cm^2^ were used as working electrode for electrochemical measurements. A saturated calomel electrode (SCE) and a platinum foil (~1 cm^2^) were used as a reference and a counter electrode (CE), respectively. All potentials were measured against the SCE (0.244 V vs. the standard hydrogen electrode, SHE). The open circuit potential (OCP) was measured during 72 h in order to establish the potential evolution of each sample. The corrosion behavior of Cu based materials was monitored using electrochemical impedance spectroscopy (EIS), after 12 h, 24 h and 72 h immersion in 0.25% *v/v* D. EIS measurements were conducted at the open circuit potential, using a 10 mV root mean-square perturbation from 100 kHz to 10 mHz. Fittings were performed with ZView software 3.6. Each measurement was repeated three times in order to verify the repeatability of results.

### 3.3. Surface Analysis

#### 3.3.1. X-ray Photoelectron Spectroscopy (XPS) Test

To further evaluate the changes on the Cu based materials surface, XPS was used to analyze the surface chemical composition of polished samples after immersion during 0 h, 2 h, 12 h, 24 h and 72 h in 0.25% *v/v* D. XPS measurements were performed by an THERMO K alpha+ spectrometer using mono-chromatised Al K_α_ radiation (1486.6 eV) controlled by the Advantage processing software. The pressure of the analysis chamber was maintained at less than 10^−7^ Pa during acquisition. No sputter cleaning was performed prior to analysis. Spectra were acquired at detection angle of 90° (normal angle with respect to the plane of the surface). For all samples, peaks were shifted according to C-C/C-H bonds of aliphatic carbon on C 1s peaks at 285 eV.

No charge compensation was needed on samples at 0 h, 2 h and 12 h. Charge compensation using an electron flood gun was used on samples at 24 h and 72 h because of the presence of oxide layer.

Pass energy was 80 eV which corresponds to Ag 3d5/2 FWHM (full width at half maximum) of 0.75 eV on sputtered silver foil.

#### 3.3.2. Time of Flight Secondary Ion Mass Spectroscopy (ToF-SIMS)

The Time of Flight-Secondary Ion Mass Spectrometry (ToF-SIMS) technique was used to perform the molecular analysis of the materials extreme surface studied in this work. The samples analyzed by ToF-SIMS method were: disinfectant solution deposited on aluminum substrate (noted D), pure copper (Cu), Former alloy and New alloy after polishing (0 h) and after 72 h of immersion in the disinfectant solution (D). The purpose of ToF-SIMS analysis was to determine the nature of the chemical compounds present at the extreme surface of the samples. The approach conducted was semi-quantitative. The measurements were performed using a ToF-SIMS IV ION-TOF instrument with a pulsed bismuth (Bi^3+^, 25 keV) primary ion source at a pressure of ~10^−9^ mbar controlled by SurfaceLab 6.7 software.

The positive and negative mass spectra of the surface chemical components were collected on a surface of 200 × 200 μm^2^, with analysis depth of 1–2 nm (the first molecular layer), in three positions. Data acquisition and post-processing were conducted using surface Lab 6.7 software. 

### 3.4. Antibacterial Tests

#### 3.4.1. Metal Preparation for Antibacterial Tests

Four types of metal samples were used during antimicrobial tests: stainless steel (SS) coupons (provide by Goodfelow) of 55 mm × 10 mm × 1 mm used as negative antimicrobial control, pure copper coupons (provide by Goodfelow) of the same dimensions and used as positive antimicrobial control, Former and New copper alloy pieces of 40 mm × 8 mm × 8 mm. As for corrosion tests, pure copper and copper alloys samples were polished at P1200 using a polishing machine, one hour before artificial aging.

#### 3.4.2. Artificial Aging of Metal Samples

As for corrosion tests prior to each test, Aniosurf Premium was diluted in deionized water at 0.25% *v/v*. Metal samples were immersed in independent D solutions during different times (0 h to 72 h). Following each immersed time, samples were collected, washed with 20 sprays of sterile deionized water, then dried by gentle stamps with paper towel before being evaluated for their antibacterial activity.

#### 3.4.3. Preparation of MRSA 

The methicillin-resistant *Staphylococcus aureus* (MRSA) strain CIP 103811 was conserved at −80 °C and thawed before use. Bacteria were spread on tryptic soy agar (TSA) and incubated at 37 °C for 24 h. For each run of the tests, a single colony was transferred in a tube containing 10 mL of tryptic soy broth (TSB) (Sigma-Aldrich, Dutscher, Brumath, France) and incubated overnight at 37 °C, with stirring. Afterward, 3 mL of the culture were transferred to an Erlenmeyer containing 50 mL of fresh TSB and this subculture was incubated for four hours at 37 °C with stirring. Then, the culture was washed three times with centrifugation at 1873 g for 5 min and addition of 10 mL of peptone water. Finally, the bacterial pellet was resuspended in peptone water to reach a concentration of 10^10^ colony forming units (CFU)/mL. 

#### 3.4.4. Antibacterial Assays

Under sterile conditions, three droplets of 10 µL containing around 10^8^ CFU were inoculated independently on the surface of each sample and incubated for two hours at room temperature. Afterward, each inoculum was harvested using a sterile swab humidified with 50 µL of peptone water. Each swab was placed in a 50 mL sterile tube containing 7.5 mL of peptone water. Tubes were ultrasonicated at 35 kHz for 2 min, then vortexed for 30 s in order to resuspend bacteria in the medium. Serial dilutions were performed and 100 µL of each dilution were plated on TSA exponentially with the easySpiral Pro (Inerscience, Saint-Nom-la-Bretèche, France). After 24 h of incubation at 37 °C, the CFU on the agar plates were enumerated with the Scan 1200 (Interscience). 

#### 3.4.5. Statistical Analysis

The non-parametric Mann–Whitney test was used to statistically compare series of biological data, using Prism software (version5, GraphPad Software, San Diego, CA, USA). The differences between series were considered as significant when *p* < 0.05.

## 4. Conclusions

Two copper alloys containing 92.5 wt% of copper were characterized by the combination of electrochemical methods, XPS and ToF-SIMS analyses and have been compared with pure copper. The addition of different alloying elements at the same copper content (Al, Zn and Si) had a major influence on the corrosion and antibacterial properties of materials. The analyses revealed that the organic adsorption compounds onto the copper surface can be additionally corroborated by the decrease in the film capacitance in the presence of D solution. Q_f_ parameter decreases for all the samples during 72 h of immersion in D solution, but the rate of reaction is different for each sample. A less important evolution of the film has been noted for the New alloy containing Zn and Si. After 72 h of immersion in D solution, the Q_f_ values of organic film are similar for all the samples. This result shows that the thickness of the formed film is the same, but the active surface is different for New alloy. This study proves that the percentage of copper alone does not determine the alloy anticorrosion and antibacterial behavior, but that the nature and quantities of minor elements are of extreme importance. Thus, the addition of zinc and silicon in low concentration made it possible to generate an active film on the surface of New alloy samples, allowing the antimicrobial properties of this new material to be maintained over time. This new alloy, more aesthetic than pure copper for the users, does not seem to interact unfavorably with the D solution over time and therefore better retains the antimicrobial power than the Former alloy. On the contrary, it reacts with this environment to form an organic film while keeping the antimicrobial power of bulk copper. The ToF-SIMS technique provides conclusive proof of sulfur presence in the D solution and that it is responsible for the loss of activity of the copper-based materials by the formation on the surface of an organic film consisting mainly of C, N, O and S (chemical elements identified in the disinfectant).

## Figures and Tables

**Table 1:** Parameters obtained by fitting the EIS-R_e_ plots shown in Figure 2b with the equivalent circuit presented in Figure 3.

Samples Name	Q_f_		R_f_ [kΩcm^2^]	Q_dl_		R_t_ [kΩcm^2^]	10^−4^χ^2^
	10^−6^Y_01_ [S sec^n1^/cm^2^]	n_f_		10^−6^Y_02_ [S sec^n2^/cm^2^]	n_dl_		
Copper 12 h	16.6	0.6	10.6	43.6	0.5	61.3	11.9
Copper 24 h	10.4	0.6	2.7	66	0.4	55	7.8
Copper 72 h	0.5	0.8	2.2	30	0.4	108	2
Former alloy 12 h	12.9	0.5	1.50	37.5	0.6	122	8.1
Former alloy 24 h	2.1	0.7	2.1	37.9	0.5	130.2	2.9
Former alloy 72 h	0.5	0.8	5	27.1	0.5	187	1.4
New alloy 12 h	3.2	0.7	4.8	34.5	0.5	83	8.7
New alloy 24 h	1.13	0.8	5.1	38.1	0.4	99.8	5.9
New alloy 72 h	0.26	0.9	3.3	49.9	0.4	75.7	2.2

## Data Availability

The data presented in this study are available upon request from the corresponding author.

## Supplementary Materials

The following supporting information can be downloaded at: https://www.mdpi.com/article/10.3390/molecules28030981/s1, Table S1: Relative intensity of positive ions: cationic surfactant and unidentified species from D solution; Table S2: Relative intensity of positive ions: metallic elements and Copper/oxygen compounds; Table S3: Relative intensity of positive ions: Aliphatic hydrocarbon species, Aromatic hydrocarbon species and Nitrogenous organic species; Table S4: Relative intensities (negative ions): sulfur and halogen species; Table S5. Proportion of chlorine chemical forms; Table S6. Proportion of sulfur chemical forms; Table S7. Relative intensity of positive ions: cationic surfactant and unidentified species from D solution; Table S8. Relative intensity of positive ions: metallic elements and Copper /oxygen compounds; Table S9. Relative intensity of positive ions: Aliphatic hydrocarbon species, Aromatic hydrocarbon species and Nitrogenous organic species; Table S10. Relative intensities (negative ions): sulfur and halogen species.

## Sample Availability

Samples of the copper alloys before and after tests are available from the authors.
