# Supplementary material for: Effects of Ageing in Disinfectant Solution on the Corrosion Resistance and Antimicrobial Behavior of Copper Alloys
Source: Molecules. 2023 Jan 18;28(3):981. doi: 10.3390/molecules28030981 (PMC9921941; doi:10.3390/molecules28030981)
Supplement: Supplementary file 1 [file molecules-28-00981-s001.zip › molecules-2100110-supplementary.pdf]

molecules

MDPI

Supplementary Material

Effects of Ageing in Disinfectant Solution on the Corrosion Resistance and Antimicrobial Behavior of Copper Alloys

Florica Simescu Lazar [1], Marius Colin [2], Gaëlle Carré [2], Nicolas Bachelard [1], Jean-Paul Chopart [1,*] and Sophie C. Gangloff [2]

[1] Laboratoire Matériaux et Ingénierie Mécanique (MATIM), UFR de Sciences Exactes et Naturelles, Université de Reims Champagne-Ardenne, 51687 Reims, France
[2] Biomatériaux et Inflammation en site Osseux (EA 4691 BIOS), SFR CAP-Santé, UFR de Pharmacie, Université de Reims Champagne-Ardenne, 51095 Reims, France
* Correspondence: jean-paul.chopart@univ-reims.fr

The supplementary materials include four tables noted S1-S10

Table S1. Elementary composition (atomic %) of the different samples: Disinfectant on silicon substrate (D/Si), Copper, Former alloy and New alloy.

Sample/elementary composition	C at%	O at%	N at%	Cl at%	S at%	Na at%	Cu at%	Al* at%	Zn at%	Si at%
**D/Si substrate**	84.4	8.5	2.9	3	0.6	0.6	-	-	-	-
**Copper 0h**	32.4	27.4	1.45	1.35	-	-	37			
**Copper 2h**	54.6	26.7	3.7	0.9	3.1	-	11.1			
**Copper 12h**	69.5	12.4	8.6	1.4	4.4	-	4			
**Copper 24h**	63.8	18.7	6.9	1	3	-	7.6			
**Copper 72h**	71.2	11.1	9.15	1.35	4.65	-	2.65			
**Former alloy 0h**	34.5	32.2	1.1	0.45	-	-	19.65	11.25		
**Former alloy 2h**	55.7	22.5	7.65	1.55	2.05	-	7.55	3.65		
**Former alloy 12h**	67.55	9.65	12.85	2.5	4.5	-	3	-		
**Former alloy 24h**	68.1	13.3	9.9	1.8	3.4	-	3.5	-		
**Former alloy 72h**	72.15	9.45	10.1	1.6	4.55	-	2.2	-		
**New alloy 0h**	39.15	29.7	1.3	0.6	-		24.0		0.9	3.9
**New alloy 2h**	61	18.35	8.4	2	3.05		5.9		0.2	1.15
**New alloy 12h**	68.95	10.7	11.55	2.0	4.25		2.6		-	-
**New alloy 24h**	72.0	9.5	11.2	1.7	3.7		1.8		-	0.3
**New alloy 72h**	72.7	8.3	12.1	2.0	3.5		1.5		-	-

*NB: The at% of Al was calculated from the Cu_{3s} peak by subtracting the contribution of the Cu_{3s} peak. This contribution was estimated by the ratio of the Cu_{3s} / $Cu_{2p3/2}$ area peaks with a pure copper.

Table S2. Proportion of chemical forms of carbon for: (a) Copper, (b) Former alloy and (c) New alloy, expressed as percentage of form relative to the total area of the peak.

Sample/Proportion of chemical forms	C-C / C-H	C-O / C-N	C=O/O-C-O	O-C=O
**Copper 0h**	53	29	12	6
**Copper 2h**	78	15	5	2
**Copper 12h**	75*	20*	4*	1*
**Copper 24h**	51	35	11	3
**Copper 72h**	86*	12*	1*	1*
**Former 0h**	63	23	9	5
**Former 2h**	66	22	8	4
**Former 12h**	60	24	14	2
**Former 24h**	71	23	5	1
**Former 72h**	88	11	-	1
**New alloy 0h**	62	26	9	3
**New alloy 2h**	75	18	4	3
**New alloy 12h**	63	22	13	2
**New alloy 24h**	49	37	12	2
**New alloy 72h**	54	23	18	5

Table S3. Proportion of chemical forms of copper on Copper, Former alloy and New alloy surface.

Samples/ chemical forms of copper	Cu 0	Cu I	Cu II	Satellite
Copper 0h	++	+	-	-
Copper 2h	-	++	++	++
Copper 12h	-	++	++	+
Copper 24h	-	+	+++	+
Copper 72h	-	+	+++	+
Former 0h	++	+	-	-
Former 2h	*	++	++	+
Former 12h	-	++	+++	+
Former 24h	-	+	+++	+++
Former 72h	-	+++	+	+
New alloy 0h	++	+	-	-
New alloy 2h	*	++	++	++
New alloy 12h	-	++	++	+
New alloy 24h	-	-	+++	+++
New alloy 72h	-	+++	++	+

* slight shoulder on the Auger peak which could correspond to the presence of a small proportion of metal shape.

Table S4. Proportion of nitrogen chemical forms for Copper, Former alloy and New alloy.

**Sample/Nitrogen chemical forms**	**398 eV**	**399-400 eV**	**~ 402 eV**
**Copper 0h**	31	69	-
**Copper 2h**	33	34	33
**Copper 12h**	35	40	26
**Copper 24h**	35	39	26
**Copper 72h**	33	39	28
**Former 0h**	48	43	8
**Former 2h**	49	44	7
**Former 12h**	34	47	19
**Former 24h**	36	45	19
**Former 72h**	33	40	27
**New alloy 0h**	31	51	18
**New alloy 2h**	39	48	13
**New alloy 12h**	37	48	15
**New alloy 24h**	30	48	22
**New alloy 72h**	34	45	21

Table S5. Proportion of chlorine chemical forms.

Sample/Chlorine chemical forms	Chlorine (198 eV)	Organic (200.5 eV)
**D solution**	100	-
**Copper 0h**	100	-
**Copper 2h**	64	36
**Copper 12h**	31	69
**Copper 24h**	31	69
**Copper 72h**	26	73
**Former 0h**	100	-
**Former 2h**	20	80
**Former 12h**	19	81
**Former 24h**	28	72
**Former 72h**	27	73
**New 0h**	100	-
**New 2h**	43	57
**New 12h**	17	83
**New 24h**	15	85
**New 72h**	15	85

Table S6. Proportion of sulfur chemical forms.

Samples name	Sulfide form (162 eV)	Sulfate and/or sulfonate forms (168-169 eV)
**D solution**	37	63
**Copper 0h**	-	-
**Copper 2h**	31	69
**Copper 12h**	42	58
**Copper 24h**	33	67
**Copper 72h**	39	61
**Former 0h**	-	-
**Former 2h**	43	57
**Former 12h**	44	56
**Former 24h**	29	71
**Former 72h**	39	61
**New 0h**	-	-
**New 2h**	40	60
**New 12h**	44	56
**New 24h**	39	61
**New 72h**	40	60

Table S7. Relative intensity of positive ions: cationic surfactant and unidentified species from D solution.

**Sample**	**N° of tests**	**Intensity of substrate**	**Cationic surfactant**						**Unidentified species from solution D**			
		^{65}Cu (100%)	$C_{12}H_{26}N$	$C_{16}H_{34}N$	$C_{18}H_{38}N$	$C_{22}H_{48}N$	452 uma	466 uma	635 uma	1139 uma	1170 uma	1202 uma
**Copper 0h**	1 to 3	1 469 364<br>1 449 800<br>1 507 234	0.45	0.24	0.22	4.2	0.14	0.62	-	-	-	-
**Copper 72h**	1 to 3	154 036<br>89 813<br>111 941	803	259.3	206.6	2573.3	109.3	9	2.56	0.31	0.54	0.1
**Former alloy 0h**	1 to 3	575 605<br>555 940<br>596 888	1.13	0.79	0.553	6.53	-	-	-	-	-	-
**Former alloy 72h**	1 to 3	95 582<br>87 247<br>103 441	1330	389	298.3	3406.66	780	23.3	2.5	0.16	0.62	0.266
**New alloy 0h**	1 to 3	1 435 064<br>1 374 332<br>1 301 584	1.33	0.35	0.27	12.66	-	-	-	-	-	-
**New alloy 72h**	1 to 3	132 125<br>124 237<br>123 896	1370	420	332	2436.6	940.3	26.66	3.8	0.3	1.4	29.33

Table S8. Relative intensity of positive ions: metallic elements and Copper /oxygen compounds.

Sample	N° of tests	Intensity of substrate	Metallic elements				Copper/oxygen compounds				
		^{65}Cu (100%)	Al	Ag*	Zn*	Si	Cu_2OH	$^{65}CuCu_3O$	$^{65}CuCu_4O$	$^{65}CuCu_4O_2$	$^{65}CuCu_5O_2$
**Copper 0h**	1 to 3	1 469 364	-	-	-	-	19.3	1.23	1.93	2.83	0.55
		1 449 800									
		1 507 234									
**Copper 72h**	1 to 3	154 036	-	-	-	-	22	-	-	-	-
		89 813									
		111 941									
**Former alloy 0h**	1 to 3	575 605	63.3	-	-	-	17	0.69	1.43	1.63	0.7
		555 940									
		596 888									
**Former alloy 72h**	1 to 3	95 582	0.74	-	-	-	10.26	-	-	-	-
		87 247									
		103 441									
**New alloy 0h**	1 à 3	1 435 064	-	-	-	5.36	21.6	1.13	2.03	2.53	0.55
		1 374 332									
		1 301 584									
**New alloy 72h**	1 à 3	132 125	-	-	-	1.16	11	-	-	-	-
		124 237									
		123 896									

*NB: Relative intensity of the positive ions corresponding to the Ag and Zn metallic elements has not identified by ToF- SIMS analyses. This shows that these elements has not found on the samples extreme surface.

Table S9. Relative intensity of positive ions: Aliphatic hydrocarbon species, Aromatic hydrocarbon species and Nitrogenous organic species.

Sample	N° of tests	Intensity of substrate	Aliphatic hydrocarbon compounds			Aromatic hydrocarbon compounds		Nitrogenous organic species		
		^{65}Cu (100%)	C_2H_5	C_3H_7	C_4H_9	C_6H_5	C_7H_7	CH_4N	C_3H_8N	$C_4H_{10}N$
**Coppe 0h**	1 to 3	1 469 364	57.6	66.33	20.66	5.96	4.4	4	193	2.6
		1 449 800								
		1 507 234								
**Copper 72h**	1 to 3	154 036	**1560**	**2740**	**1343**	**112**	**72**	**105.3**	**3020**	**245.6**
		89 813								
		111 941								
**Former 0h**	1 to 3	575 605	265.7	329.3	109.66	26.66	26.66	11.33	113	6.96
		555 940								
		596 888								
**Former 72h**	1 to 3	95 582	**2180**	**3360**	**2006.66**	87.66	87.66	163.33	**3663.3**	350.66
		87 247								
		103 441								
**New 0h**	1 to 3	1 435 064	103	120	49	10.6	9.5	5.13	25.33	3.033
		1 374 332								
		1 301 584								
**New 72h**	1 to 3	132 125	1950	2403.3	1543.33	129.33	81.66	144.66	**2663.33**	321.66
		124 237								
		123 896								

Table S10. Relative intensities (negative ions): sulfur and halogen species.

**Sample**	**N° of tests**	**Intensity of substrate**	**Sulfur species**								**Nitrites/Nitrates**		**Halogen**
		^{63}Cu (100%)	S	HS	S_2	CSN	SO_2	SO_3	SO_4H	S_2O_8H	NO_2	NO_3	Cl
**Copper 0h**	1 à 3	1 469 364	5	9.23	NM	2.5	52	315.33	60	NM	15.66	11.3	812.66
		1 449 800											
		1 507 234											
**Copper 72h**	1 à 3	154 036	1096.6	1763.33	77.33	280.33	2466.66	16200	13200	880	NM	NM	5990
		89 813											
		111 941											
**Former alloy 0h**	1 à 3	575 605	100.33	189	-	174.66	180.66	514.33	292.66	-	109.66	152.66	648.33
		555940											
		596888											
**Former alloy 72h**	1 à 3	95582	1493.33	2413.33	138.33	1024.66	3543.33	24633	9153.33	1533.33	114.33	376	21333.33
		87247											
		103441											
**New alloy 0h**	1 à 3	1 435 064	33.66	44.66	-	25.33	53	158	131.33	8.2	42.66	58.66	420.66
		1 374 332											
		1 301 584											
**New alloy 72h**	1 à 3	132 125	1136.66	1820	78.66	757.66	2633.33	17200	6380	1650	95.98	290.33	17000
		124 237											
		123 896											
